# Epidemiology of virus-induced wheezing/asthma in children

**DOI:** 10.3389/fmicb.2013.00391

**Published:** 2013-12-16

**Authors:** Yuzaburo Inoue, Naoki Shimojo

**Affiliations:** Department of Pediatrics, Graduate School of Medicine, Chiba UniversityChiba, Japan

**Keywords:** wheezing, viral respiratory infection, cohort study, interferon, respiratory syncytial virus, rhinovirus

## Abstract

Wheezing is a lower respiratory tract symptom induced by various viral respiratory infections. Epidemiological studies have revealed the phenotypes of wheezing in early childhood which have different risk factors for the development of asthma among school age children. The major viral species causing wheezing in children include respiratory syncytial virus, rhinovirus, human metapneumovirus and influenza viruses. It has been shown that the impact on the development of asthma is different between those virus species. Moreover, recent studies have also focused on the interaction between virus infection and other risk factors in the development of asthma, such as genetic factors or allergic sensitization. In this review, we summarize the previous findings and discuss how clinicians can effectively intervene in these viral infections to prevent the development of asthma.

## INTRODUCTION

Wheezing is a lower respiratory tract symptom induced by various viral respiratory infections. It is in common in children, with approximately one-third of the children having at least one wheezing episode by age nine. However, about 1 to 2% of affected infants need to be hospitalized due to respiratory distress. Moreover, although the condition is transient in the majority of cases, some children develop recurrent wheezing and are diagnosed to have asthma when they reach school age. In those children, the virus-induced wheezing in early childhood may be associated with the subsequent development of recurrent wheezing and/or asthma in connection to the pathology of asthma, including chronic airway inflammation, thus leading to airway hyperresponsiveness and airway remodeling.

Epidemiological studies are therefore considered to be important for clarifying which populations are at risk for developing virus-induced wheezing accompanied with other severe symptoms, recurrent wheezing and especially asthma. Moreover, recent studies have also focused on the interaction between virus infection and other risk factors for the development of asthma, such as genetic factors or allergic sensitization.

In this review, we summarize the previous findings and discuss how clinicians can effectively intervene in these viral infections to prevent the development of asthma.

## PHENOTYPES OF VIRUS-INDUCED WHEEZING

Birth cohort studies have been conducted to clarify the natural history of wheezing in early childhood and to assess the risk factors for the development of wheezing and subsequent asthma. The first large prospective study focusing on the wheezing history was performed by the Tucson Children’s Respiratory Study group (Halonen et al., 1999; Sherrill et al., 1999; Stein et al., 1999; Taussig et al., 2003; Morgan et al., 2005). They followed 828 infants until the age of 6 years and identified four different patterns of wheezing in early childhood (never wheeze, transient early wheeze, late-onset wheeze, and persistent wheeze) on the basis of clinical observations. The “never wheeze” phenotype (51.5% of the cohort) was defined as children with no episodes of wheezing during the first 6 years of the life. The “transient early wheeze” phenotype (19.9% of the cohort) was defined as children having at least one lower respiratory tract illness with wheezing during the first 3 years of the life, but no wheezing at 6 years of age. The children with this phenotype had a diminished airway function both before the age of 1 year and at the age of 6 years, were more likely than the other children to have mothers who smoked but not mothers with asthma, and did not have elevated serum IgE levels or skin-test reactivity. The “late-onset wheeze” phenotype (15.0% of the cohort) was defined as children having no wheezing before the age of the 3 years, but having wheezing at 6 years of age. The “persistent wheeze” phenotype (13.7% of the cohort) was defined as children having wheezing both before 3 years of age and at 6 years of age. The children of this phenotype were more likely than the “never wheeze” children to have mothers with a history of asthma, to have elevated serum IgE levels and normal lung function in the first year of life, and to have elevated serum IgE levels and diminished airway function at 6 years of age. Interestingly, these phenotypes have been shown to be associated with different risk factors for the number of encountered viral infections in early childhood (Kusel et al., 2007) and the development of asthma (Taussig et al., 2003; Stein and Martinez, 2004).

Birth cohort studies from Europe using latent class analysis identified more complicated wheezing phenotypes, including an intermediate-onset wheezing phenotype. A population-based birth cohort study of 6265 children in the United Kingdom (the ALSPAC study) identified six wheezing phenotypes in childhood, from birth to age 7 years, and demonstrated that these phenotypes differed in the atopy prevalence and lung function levels at 7–8 years of age (Henderson et al., 2008). Another multicenter birth cohort study of 2810 children in the Netherlands (the PIAMA study) also identified five wheezing phenotypes in childhood from birth to age 8 years. Interestingly, the wheezing phenotypes identified by the two birth cohort studies were comparable (Savenije et al., 2011).

## VIRAL SPECIES CAUSING WHEEZING

The major viral species causing wheezing in children include respiratory syncytial virus (RSV), rhinovirus (HRV), human metapneumovirus (hMPV) and influenza viruses.

### RSV

Respiratory syncytial virus is a medium-sized negative-stranded RNA virus of the family *Paramyxoviridae*, which causes respiratory infections mainly in children. Interestingly, the clinical symptoms of RSV infection in infancy and early childhood are extremely variable. Most infants experience an RSV infection before 3 years of age (Ruuskanen and Ogra, 1993), normally escaping with only upper respiratory diseases, whereas approximately 1–2% of them require hospitalization because of severe RSV bronchiolitis (Green et al., 1989; Stretton et al., 1992). This is particularly common in those who are premature or who have chronic lung disease or congenital heart disease. Recently, a humanized monoclonal antibody designed to provide passive immunity against an epitope in the A antigenic site of the F protein of RSV has been widely used for the prophylaxis of severe RSV lower respiratory infection in those children.

### HRV

Rhinovirus is a small-sized positive-strand RNA virus of the family *Picornaviridae*, which is well known as the predominant cause of the common cold. Because of the development of PCR techniques, it has been recognized that HRVs cause not only upper respiratory infections, but also lower respiratory infections or asthma exacerbation. HRVs consist of over 100 types classified into one of three species (A, B, and C) according to the phylogenetic sequence criteria. HRV C (HRV-C) is a recently classified group and has been shown to be associated with severe asthma attacks more frequently than other groups of HRV. The prevalence of HRV-associated wheezing increases by age, and it is significantly more common in children with recurrent wheezing episodes than in first-time wheezers in age categories of <24 and <36 months (Jartti et al., 2009).

### HMPV

The hMPV is a medium-sized negative-stranded RNA virus of the family *Paramyxoviridae*, which was recently discovered (van den Hoogen et al., 2001), the clinical course of which resembles RSV infection. Similar to RSV, it has been reported that hMPV infection was associated with wheezing among children younger than 3 years, especially during the winter, while hMPV was not significantly associated with wheezing requiring hospitalization among children 3 years of age and older (Williams et al., 2005).

### INFLUENZA VIRUSES

Influenza viruses are a medium-sized negative-stranded RNA virus of the family of *Orthomyxoviridae.* Influenza viruses cause severe lower respiratory tract complications, such as bronchitis or pneumonia. In addition, influenza is significantly associated with wheezing during the winter among children younger than 3 years of age although the detection percentage of the influenza virus is lower than that of RSV (Heymann et al., 2004).

## RISK FACTORS FOR THE DEVELOPMENT OF VIRUS-INDUCED WHEEZING

### BEHAVIORAL OR ENVIRONMENTAL FACTORS

In the Tucson study, it was reported that breast-feeding at early infancy for at least 1 month was associated with lower rates of virus-induced wheezing during the first 4 months of the life (Wright et al., 1989). However, the results of the subsequent studies have been conflicting. A meta-analysis study finally showed that there was no association between any or exclusive breast feeding and wheezing illness (Brew et al., 2011).

Infants exposed to more children at home or day care experienced more frequent wheezing when they were 2 years old, but less frequent wheezing from years eight through year thirteen. Therefore, although exposure to children at home or in day care during infancy increased wheezing in early life, it appears to be protective against the development of frequent wheezing in school age children (Ball et al., 2000).

### HOST IMMUNOLOGICAL FEATURES

Interferon (IFN) secretion is important in the clearance of viral pathogens. Therefore, IFN deficiency has been supposed to lead to lower respiratory viral infections. There are three types of interferons: Type I (IFN-α/β), Type II (IFN-γ) and Type III (IFN-λ). It was shown that low IFN-γ production in cord bloods (Copenhaver et al., 2004) or PBMCs in the first year of life (Stern et al., 2007) was a risk factor for wheezing during childhood, in addition to a risk factor for the development of asthma and allergies (Tang et al., 1994). It has recently been clarified that the deficiency of IFN production is related to atopy. It was reported that allergic asthmatic children had an impaired HRV-induced IFN-α and IFN-λ1 production that correlated with an increased FcεRI expression on plasmacytoid dendritic cells in PBMCs, which were reduced by FcεRI cross-linking (Durrani et al., 2012). In addition, it was reported that bronchial epithelial cells from asthmatic individuals produced less IFN-β in response to HRV, leading to impaired apoptosis and increased HRV replication (Wark et al., 2005). Interestingly, it was revealed that allergic sensitization precedes HRV-induced wheezing, but the converse is not true (Jackson et al., 2012). The results of that study suggested that allergic sensitization can lead to more severe HRV-induced lower respiratory illnesses, which is considered to be a risk factor for the subsequent development of asthma.

### GENETIC FACTORS

There have been many genetic risk factors reported to be associated with the development of RSV bronchiolitis. Two large scale genetic association studies were performed using a candidate gene approach (Janssen et al., 2007; Siezen et al., 2009). They analyzed 384 single-nucleotide polymorphisms (SNPs) in 220 candidate genes involved in the airway mucosal responses, innate immunity, chemotaxis, adaptive immunity, and allergic asthma. They found that SNPs in genes of the innate immune responses (the transcriptional regulator Jun, alpha interferon, IFN-α, nitric oxide synthase and the vitamin D receptor) are important for determining the susceptibility to RSV bronchiolitis in term children. As RSV is recognized by Toll-like receptor (TLR) 4, SNPs in the genes of molecules related to TLR4 signaling have also been studied (Tal et al., 2004; Inoue et al., 2007).

In contrast, the genetic factors related to the development of HRV-induced wheezing are less well known (Helminen et al., 2008; Caliskan et al., 2013). However, the 17q21 variants, which were found to be related to childhood-onset asthma in a genomewide association study (Moffatt et al., 2007), were associated with HRV wheezing illnesses in early life, but not with RSV wheezing illnesses (Caliskan et al., 2013).

## ASSOCIATION BETWEEN VIRUS-INDUCED WHEEZING AND THE DEVELOPMENT OF ASTHMA, AND EFFECTIVE TYPES OF INTERVENTION TO PREVENT THE SUBSEQUENT DEVELOPMENT OF ASTHMA

It is still unclear whether lower respiratory viral infections are causal factors, or instead serve as indicators, of a predisposition to asthma. Moreover, recent studies have indicated that the impact on the development of subsequent recurrent wheezing or asthma is different between virus species.

It was reported that infant birth approximately 4 months before the winter virus peak, which is the peak of bronchiolitis hospitalizations for that winter season, carried the highest risk for the development of asthma, thus suggesting that a lower respiratory infection with winter viruses, including RSV, in early childhood may be an important factor in the development of asthma (Wu et al., 2008). In a birth cohort study, Sigurs et al. (2010) followed 47 children aged <1 year hospitalized with RSV lower respiratory infection (RSV group) and 93 age- and gender- matched controls (Control group) for 18 years. They found that the RSV group had an increased prevalence of asthma/recurrent wheezing, clinical allergy and sensitization to perennial allergens, compared to the Control group (Sigurs et al., 2010). Meanwhile, it was shown that RSV prophylaxis using Palivizumab, a humanized monoclonal antibody against the RSV fusion protein that prevents severe RSV lower respiratory infection, in non-atopic children decreased the relative risk of recurrent wheezing by 80%, but did not have any effect in infants with an atopic family history (Simoes et al., 2010). These results suggest that RSV predisposes to recurrent wheezing via an atopy-independent mechanism.

Rhinovirus has been implicated as an important pathogen in asthma pathogenesis due to the improvement of PCR for HRV detection. In the Childhood Origins of ASThma (COAST) cohort, HRV in nasal lavage samples were evaluated by PCR. They found that, by age 3 years, wheezing in those with HRV-positive samples (OR, 25.6) was more strongly associated with asthma at age 6 years than aeroallergen sensitization (OR, 3.4; Jackson et al., 2008). As IFN deficiency is related to both atopy and the susceptibility to HRV infection, the inhalation of IFN by HRV-infected children with risk factors for asthma might thus help to prevent the development of asthma.

Recently, pandemic H1N1 influenza virus has been reported to increase the risk of lower respiratory tract complications including asthma attack, pneumonia, and atelectasis even in atopic children without any history of either an asthma attack or asthma treatment, compared to the seasonal influenza virus (Hasegawa et al., 2011). This observation suggests that the pandemic H1N1 influenza virus may be a strong risk factor contributing to the development or exacerbation of asthma.

## LIMITATIONS OF EPIDEMIOLOGY STUDIES

The correct diagnosis of individual viral infections is necessary for assessing which virus infection is important for the development of wheezing or the subsequent development of asthma. The principal diagnostic methods for respiratory viruses are virus culture, serology, immunofluorescence/antigen detection, and nucleic acid/PCR-based tests (Tregoning and Schwarze, 2010).

Although virus culture proves that the virus detected in clinically obtained samples is able to infect human cells, viral culture is time-consuming and is not appropriate for analyzing many samples in epidemiological studies. Viral serologic testing is also time-consuming, and generally requires at least two rounds of blood sampling, because viral serological testing can diagnose infections by an increase of a virus-specific antibody in the blood, which usually takes 2 weeks to develop. Most previous epidemiological studies thus evaluated viral infections by immunofluorescence/antigen detection or nucleic acid/PCR-based tests. Antigen detection is based on the use of virus-specific monoclonal antibodies. There are a variety of diagnostic test kits that use nasopharyngeal aspirate, nasopharyngeal wash or nasal swab specimens as the test material, and detect viral antigen by using either a conjugated enzyme or fluorescence. Immunofluorescence/antigen detection is appropriate for epidemiological studies because it is convenient, cheap and possible to use when handling for many samples. However, there is a limitation to the species of target viruses. Nucleic acid tests are significantly more sensitive than the other methods, and are now being multiplexed, allowing for the rapid detection of many viruses concurrently. The PCR method has greatly increased the recovery rates of viruses (Johnston et al., 1995; Rakes et al., 1999). However, the PCR-based diagnosis of viruses, especially HRV, may not necessarily indicate that the virus is causing the observed disease, because virus RNAs can be detected by PCR for several weeks after the onset of clinical symptoms (van Benten et al., 2003; Jartti et al., 2004; Wright et al., 2007).

Another limitation in epidemiological studies assessing wheezing/wheezes in early childhood is the difficulty of diagnosing these conditions in young children based on the clinical assessment of symptoms by both guardians and clinicians. It was reported that the parents of children aged 4 months to 15 years and clinicians agreed on only 45% of occasions that the patient was wheezy or had asthma (Cane et al., 2000), thus suggesting that epidemiological studies using symptom records kept by guardians may sometimes lead to a wrong conclusion. Moreover, it has been shown that even specialists might not always correctly recognize wheezing (Bisgaard and Bonnelykke, 2010).

## FUTURE PROSPECTIVE

To our knowledge, previous epidemiological studies regarding the association between viral infections and wheezing/asthma did not evaluate all other risk factors for the development of wheezing/asthma in childhood, including behavioral factors, environmental factors, host immunological features and genetic factors. Large birth cohort studies evaluating viral infections and these factors in childhood could thus better elucidate the impact of viral infections on the development of wheezing/asthma. However, epidemiological studies only reveal an association, but not a causal relationship between some viral infections and the development of wheezing or asthma. Therefore, in the future, intervention trials with preventative intervention or therapies on a specific virus would be needed to clearly identify when and how clinicians should intervene in such viral infections to thereby prevent the development of wheezing or asthma.

## CONCLUSION

Virus-induced wheezing is not only a burden in early childhood, but also may be one of causes or signs of childhood asthma. Therefore, clarifying the risk factors for virus-induced wheezing in epidemiological studies can and have provided clues about the pathogenesis of asthma. Further studies are needed to clarify which virus(es) in which population should be the major target of early intervention for preventing the subsequent development of asthma.

## Conflict of Interest Statement

The authors declare that the research was conducted in the absence of any commercial or financial relationships that could be construed as a potential conflict of interest.
